# Mitochondrial Genome of the Freshwater Jellyfish *Craspedacusta sowerbyi* and Phylogenetics of Medusozoa

**DOI:** 10.1371/journal.pone.0051465

**Published:** 2012-12-11

**Authors:** Hong Zou, Jin Zhang, Wenxiang Li, Shangong Wu, Guitang Wang

**Affiliations:** 1 The Key Laboratory of Aquatic Biodiversity and Conservation of the Chinese Academy of Sciences, Wuhan, Hubei Province, P.R. China; 2 Institute of Hydrobiology, Chinese Academy of Sciences, Wuhan, Hubei Province, P.R. China; 3 Graduate School of the Chinese Academy of Sciences, Beijing, P.R. China; University of Veterinary Medicine Hanover, Germany

## Abstract

The 17,922 base pairs (bp) nucleotide sequence of the linear mitochondrial DNA (mtDNA) molecule of the freshwater jellyfish *Craspedacusta sowerbyi* (Hydrozoa,Trachylina, Limnomedusae) has been determined. This sequence exhibits surprisingly low A+T content (57.1%), containing genes for 13 energy pathway proteins, a small and a large subunit rRNAs, and methionine and tryptophan tRNAs. Mitochondrial ancestral medusozoan gene order (AMGO) was found in the *C. sowerbyi*, as those found in *Cubaia aphrodite* (Hydrozoa, Trachylina, Limnomedusae), discomedusan Scyphozoa and Staurozoa. The genes of *C. sowerbyi* mtDNA are arranged in two clusters with opposite transcriptional polarities, whereby transcription proceeds toward the ends of the DNA molecule. Identical inverted terminal repeats (ITRs) flank the ends of the mitochondrial DNA molecule, a characteristic typical of medusozoans. In addition, two open reading frames (ORFs) of 354 and 1611 bp in length were found downstream of the large subunit rRNA gene, similar to the two ORFs of *ORF314* and *polB* discovered in the linear mtDNA of *C. aphrodite,* discomedusan Scyphozoa and Staurozoa. Phylogenetic analyses of *C. sowerbyi* and other cnidarians were carried out based on both nucleotide and inferred amino acid sequences of the 13 mitochondrial energy pathway genes. Our working hypothesis supports the monophyletic Medusozoa being a sister group to Octocorallia (Cnidaria, Anthozoa). Within Medusozoa, the phylogenetic analysis suggests that Staurozoa may be the earliest diverging class and the sister group of all other medusozoans. Cubozoa and coronate Scyphozoa form a clade that is the sister group of Hydrozoa plus discomedusan Scyphozoa. Hydrozoa is the sister group of discomedusan Scyphozoa. Semaeostomeae is a paraphyletic clade with Rhizostomeae, while Limnomedusae (Trachylina) is the sister group of hydroidolinans and may be the earliest diverging lineage among Hydrozoa.

## Introduction

Characterization of mitochondrial DNA (mtDNA) genomes in cnidarians has become an increasing interest in evolutionary and phylogenetic studies. The phylum Cnidaria is divided into two large clades, Medusozoa and Anthozoa, the former of which contains four classes, including Hydrozoa, Cubozoa, Scyphozoa, and Staurozoa [1]. Species in the Medusozoa have a medusoid body form in their life cycle, as different from those in the Anthozoa, and a linear mtDNA molecule in contrast to a single circular molecule of 14 to 20 kilobases in metazoans [2], [3], [4], [5]. However, most available data on cnidarian mtDNA sequences are derived from species in the Anthozoa, with the availability of more than 40 complete mtDNA sequences in public databases, which shows a degree of similarity to metazoan mtDNA genomes in being a circular structure [6]. Meanwhile, it seems likely that there is an increasing amount of data on mtDNA genomes in medusozoans. So far, 26 linear mtDNA genomes, although not all fully complete, have been reported from species of all four classes in the Medusozoa [7], for example, *Hydra oligactis* [8], *H. vulgaris*, and *H. magnipapillata* [9] in Hydrozoa, *Alatina moseri* [10] in Cubozoa, *Lucernaria janetae* [7] in Staurozoa, and *Aurelia aurita* in Scyphozoa [11]. Interestingly, the linear mtDNA can be divided into two to eight molecules [7], [9], [10]. Kayal et al. (2012) firstly examined linear mtDNA in Medusozoa and reconstructed the evolutionary history of linear mtDNA in medusozoans by analyzing the mitochondrial genome structures [7]. The dramatic feature of two open reading frames (ORFs), *polB* and *ORF314*, was identified in mtDNA sequences of cubozoan, scyphozoan, staurozoan, and trachyline hydrozoan, which was thought to be remnants of a linear plasmid that invaded the mitochondrial genomes of the last common ancestor of Medusozoa and should be responsible for the linearity [7], [11]. However, hydroidolinan hydrozoans have lost the two ORFs and they instead have duplicated *cox1* at each end of their mitochondrial chromosome(s) [7], [8], [9].

However, very few nucleotide sequences can be found from *Craspedacusta* in public databases, such as in GenBank. The nucleotide sequences referred to *Craspedacusta* mtDNA comprise only several *cox1* and *rnl* sequences. Recently, a nearly complete mitochondrial genome sequence of *Cubaia aphrodite* (Hydrozoa, Trachylina, Limnomedusae) was reported [7]. *C. aphrodite*, living in marine water, belongs to the same order, Limnomedusae, as freshwater jellyfish *Craspedacusta sowerbyi*. Though with sequence of the identical inverted terminal repeats (ITRs) and partial *polB* being undetermined, its mitogenome was thought to possesses characteristically the mitochondrial ancestral medusozoan gene order (AMGO), as reported in scyphozoans and staurozoans [7], [11]. But, the mitochondrial genome organizations in non-trachyline hydrozoans contain one or several linear molecules, thus exhibiting a degree of variation, with probably the presence of other three gene orders [7], [8], [9]. Mitochondrial genome organizations in the Hydrozoa may be thus much more variable than in other medusozoans. The understanding of their mtDNA genomes may provide necessary information to reveal the diversity of mtDNA genome organization and their evolution. More mitogenomes from trachyline hydrozoans are certainly needed to test whether AMGO is typical of this group or whether other gene orders exist.

Freshwater jellyfish are frequently reported, with more than 20 species of freshwater jellyfish in the world [12]. Among them, *Craspedacusta sowerbyi* (Hydrozoa, Trachylina, Limnomedusae) is the most widely distributed species [13]. It is considered that this species is originated from the Yangtze River area in China [13], [14], but has been dispersed to many freshwater habitats in the world [15], [16], [17], [18], [19], [20]. The hydrozoan has two adult forms, the fixed polyp, and the free-living medusa, with the latter being the sexual form, occurring sporadically in different season of the year, although more commonly during summer in temperate regions [18]. It was thus considered possible to obtain samples of *C. sowerbyi* in China in order to examine its mtDNA genome [21]. In this report, we presented the complete sequence of the mitochondrial genome of *C. sowerbyi*, which represents the first complete mitochondrial genome for species in the Trachylina. Furthermore, we used this sequence together with other reported sequences of medusozoan mtDNA to analyze the phylogenetic relationship within the Cnidaria, and especially within the Medusozoa.

## Results

### Genome Structure, Organization, and Nucleotide Composition

**Figure 1:**
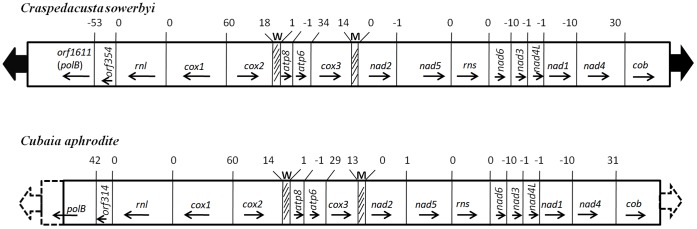
Maps of the mitochondrial genomes of *C. sowerbyi* and *C. aphrodite*. Protein and ribosomal genes (large open boxes) are abbreviated as presented in the text; tRNA genes (small hatched boxes) are identified by the one-letter code for their corresponding amino acid. Intergenic regions greater than 100 bp are shown by shaded boxes; ITR shown by two large arrows. Arrows within or under each box show the direction of transcription. Positive numbers at gene boundaries indicate the number of intergenic nucleotides; negative numbers indicate the number of overlapping nucleotides. The dash lines mean that the sequence of this part is not determined.

The sequence of a 17,922 bp segment was cloned from *C. sowerbyi*, which contains 13 known mitochondrial protein coding genes, two tRNAs, a large and a small subunit rRNAs, and two putative ORFs (Fig. 1). The genes in *C. sowerbyi* mitochondrial genome were arranged into two clusters of opposite transcriptional orientations. Transcription proceeded toward the ends of the DNA molecule, with 15 genes transcribed in one direction, and two known genes (*cox1* and *rnl*) and two putative ORFs in the other. The change in the transcriptional polarity occurred between *cox1* and *cox2*, where the longest non-coding intergenic region of 60 bp was located. The gene arrangement in *C. sowerbyi* mtDNA was consistent with AMGO, which was identical to that in trachyline *C. aphrodite* (Fig. 1), discomedusan Scyphozoa and Staurozoa, but different from those of all nontrachyline hydrozoan species [7].

Interestingly, the nucleotide composition of *C. sowerbyi* mtDNA showed a small bias towards low A+T content (being 57.1%), when compared with those in the trachyline *C*. *aphrodite* (69%), other hydrozoans (>75%), and in general, metazoans (>70%) [7], [22]. The protein coding genes had the lowest A+T content (55.0%), while the rRNA genes showed the highest A+T content (62.7%) in the *C. sowerbyi* mtDNA (Table 1). The lowest value for A+T content was observed at the third codon positions (47.1%) in the protein coding genes, whilst in other cnidarians, A+T content at the third codon positions was generally the highest, compared with other two codon positions [9]. The discrepancy of A+T content between rRNA genes and third codon positions is contradictory to the deduction by Voigt et al., that A+T content in rRNA genes was generally correlated with the usage of A and T at third codon positions in all Cnidaria [9].

**Table 1:** Nucleotide composition data for different groups of genes, ORFs, and non-coding regions in *C. sowerbyi* mtDNA.

	Full sequence	Coding sequences		ORFs	rRNA -genes	tRNA -genes	Intergenic
		Total	3rd positions				
%G	25.33	20.7	20.6	24.2	19.5	21.1	16.6
%A	21.26	22.9	22.6	24.7	29.8	28.9	29.3
%T	31.82	32.1	24.5	34.5	32.9	30.3	32.5
%C	21.59	24.3	32.4	16.6	17.8	19.7	32.7
%A+T	57.14	55.0	47.1	59.2	62.7	59.2	61.8
Total (bp)	17922	12051	4017	1965	2812	142	157

### Protein-coding Genes

Genes coding for proteins involved in respiration and oxidative phosphorylation (*atp6*, *atp8*, *cob*, *cox1*–*3*, *nad1*–*6*, and *nad4L*) in *C. sowerbyi* mtDNA were similar in size to their homologues in other medusozoans. *atp8*, *nad2*, and *nad6* were the least conserved genes at the inferred amino acid level, showing only 21 to 64% sequence identity between *C. sowerbyi* and other representatives of Medusozoa; but, this percentage goes up to 92% in *cox1*, the most conserved gene (Table 2). Comparisons of thirteen energy pathway protein genes and 4 RNA genes in *C. sowerbyi* with their homologues in other medusozoans showed that these genes shared most nucleotide sequences with *C. aphrodite* (Table 2).

**Table 2:** Comparison of the mitochondrial protein coding genes and RNA genes of the freshwater jellyfish *C. sowerbyi* (*C.s*), the jellyfish *A. aurita* (*A.a*), the hydrozoan *H. oligactis* (*H.o*), cubozoan *A. moseri* (*A.m*), and the trachyline *C. aphrodite* (*C.a*).

Genes	Number of encoded nucleotides^a^					Percent sequence identity			
	*C.s*	*A.a*	*H.o*	*A.m*	*C.a*	*C.s*/*A.a*	*C.s*/*H.o*	*C.s/A.m*	*C.s/C.a*
*Atp6*	705	705	702	711	702	69%	46%	53%	80%
*Atp8*	207	204	207	198	204	30%	21%	33%	56%
*Cob*	1185	1140	1140	1146	1143	68%	67%	71%	83%
*Cox1*	1566	1581	1575	1566	1563	82%	78%	82%	92%
*Cox2*	738	726	756	732	738	65%	57%	68%	84%
*Cox3*	786	786	786	783	783	76%	58%	78%	87%
*Nad1*	999	972	990	960	993	65%	62%	62%	80%
*Nad2*	1350	1320	1305	1338	1350	34%	30%	32%	50%
*Nad3*	357	360	348	339	354	67%	59%	54%	72%
*Nad4*	1461	1443	1458	1443	1458	55%	55%	46%	73%
*Nad4L*	309	303	300	282	297	57%	56%	57%	80%
*Nad5*	1833	1818	1833	1821	1830	51%	52%	49%	70%
*Nad6*	564	573	558	543	561	38%	37%	34%	64%
*rns*	995	960	977	893	994	63%	68%	57%	76%
*rnl*	1817	1817	1745	1679	1761	36%	39%	36%	72%
*Trn-M*	71	70	71	70	71	45%	68%	64%	93%
*Trn-W*	71	71	70	nd	71	45%	75%	nd	88%

“nd” means not determined.

a Data for *A. aurita* are from Shao et al. [10], for *H. oligactis* are from Kayal and Lavrov [8], for *A. moseri* are from Kayal et al. [7], for *C. aphrodite* are from Kayal et al. [7].

Two overlapping ORFs of 354 and 1611 nucleotides have been found downstream of *rnl*, close to the end of the *C. sowerbyi* linear mtDNA molecule. Similar ORFs were found at one end of the mtDNA molecule in all species of Staurozoa, discomedusan Scyphozoa, and the trachyline hydrozoan *C. aphrodite*, and also found on a separate mitochondrial chromosome in Cubozoa [7], [10]. The deduced amino acid sequence of *ORF354* shared the most sequence elements, but with a low level of identity (only 32%) with the poorly conserved analogue *ORF314* from *C. aphrodite*, which may act as terminal protein (TP), as suggested by Kayal et al. [7]. The deduced amino acid sequence of *ORF1611* displayed extensive sequence similarity with the family B DNA polymerases, and this ORF has been identified as *polB*. The deduced POLB containing domains involved in the 3′-5′exonuclease (Exo I to III) and polymerase (Pol I to Pol V) activities was the longest inferred POLB sequence reported for cnidarians. As discovered in the linear mtDNA of some other medusozoans, the deduced POLB shared extremely low similarity with any other species, even with *C. aphrodite* [7], [11].

Analyses of the codon usage among the 13 energy pathway protein genes and, separately, in the two ORFs were shown in Table 3. This table was compiled based on a minimally modified genetic code, with TGA = tryptophan being the only deviation deduced for mitochondrial protein synthesis in *C. sowerbyi*. Among the 13 energy pathway protein genes, only one codon, CGG, was never used, and all protein-coding genes were assumed to have complete termination codons (either TAA or TAG). All protein-coding genes in *C. sowerbyi* mtDNA started with a conventional ATG initiation codon. The proportion of GC-rich amino acids (the “GARP” amino acids: glycine, alanine, arginine, and proline) was 19.4%. Considering the dramatic high GC content (45%) of mitochondrial protein genes in *C. sowerbyi* mtDNA, this result seems not to be consistent with the previous study that a strong correlation was found between nucleotide composition and the proportion of the “GARP” amino acids [7]. The reason may be that in *C. sowerbyi* mtDNA the high GC content of the coding sequence appears largely at the third codon positions, whereas in other reported cnidarians G or C preferred to appear at the first or second codon positions that always determine the kind of amino acid to be coded for.

**Table 3:** Codon usage among the 13 energy pathway protein genes and Two ORFs.

		*C.s* *	ORFs#			*C.s* *	ORFs#			*C.s* *	ORFs#			*C.s* *	ORFs#
Phe	TTT	109	10	Ser	TCT	93	9	Tyr	TAT	75	12	Cys	TGT	30	3
	TTC	235	30		TCC	60	13		TAC	84	19		TGC	12	3
Leu	TTA	98	19		TCA	26	11	TER	TAA	8	1	Trp	TGA	80	4
	TTG	148	9		TCG	48	8		TAG	5	0		TGG	24	0
Leu	CTT	77	9	Pro	CCT	53	6	His	CAT	46	14	Arg	CGT	11	3
	CTC	114	16		CCC	60	19		CAC	59	15		CGC	5	1
	CTA	82	12		CCA	16	2	Gln	CAA	57	14		CGA	22	5
	CTG	79	10		CCG	18	3		CAG	28	7		CGG	0	1
Ile	ATT	72	12	Thr	ACT	75	9	Asn	AAT	41	12	Ser	AGT	37	5
	ATC	172	26		ACC	84	13		AAC	76	22		AGC	50	7
	ATA	98	18		ACA	17	3	Lys	AAA	52	45	Arg	AGA	49	23
Met	ATG	141	14		ACG	51	4		AAG	45	16		AGG	13	4
Val	GTT	65	5	Ala	GCT	95	2	Asp	GAT	45	5	Gly	GGT	57	5
	GTC	138	8		GCC	104	7		GAC	49	16		GGC	24	2
	GTA	50	8		GCA	44	8	Glu	GAA	71	28		GGA	130	7
	GTG	76	7		GCG	34	4		GAG	44	8		GGG	56	13

* Codon usage among the 13 energy pathway protein genes of *C. sowerbyi*;

# Codon usage of the genes *ORF1611* and *ORF354* of *C. sowerbyi.*

### RNA Genes

**Figure 2:**
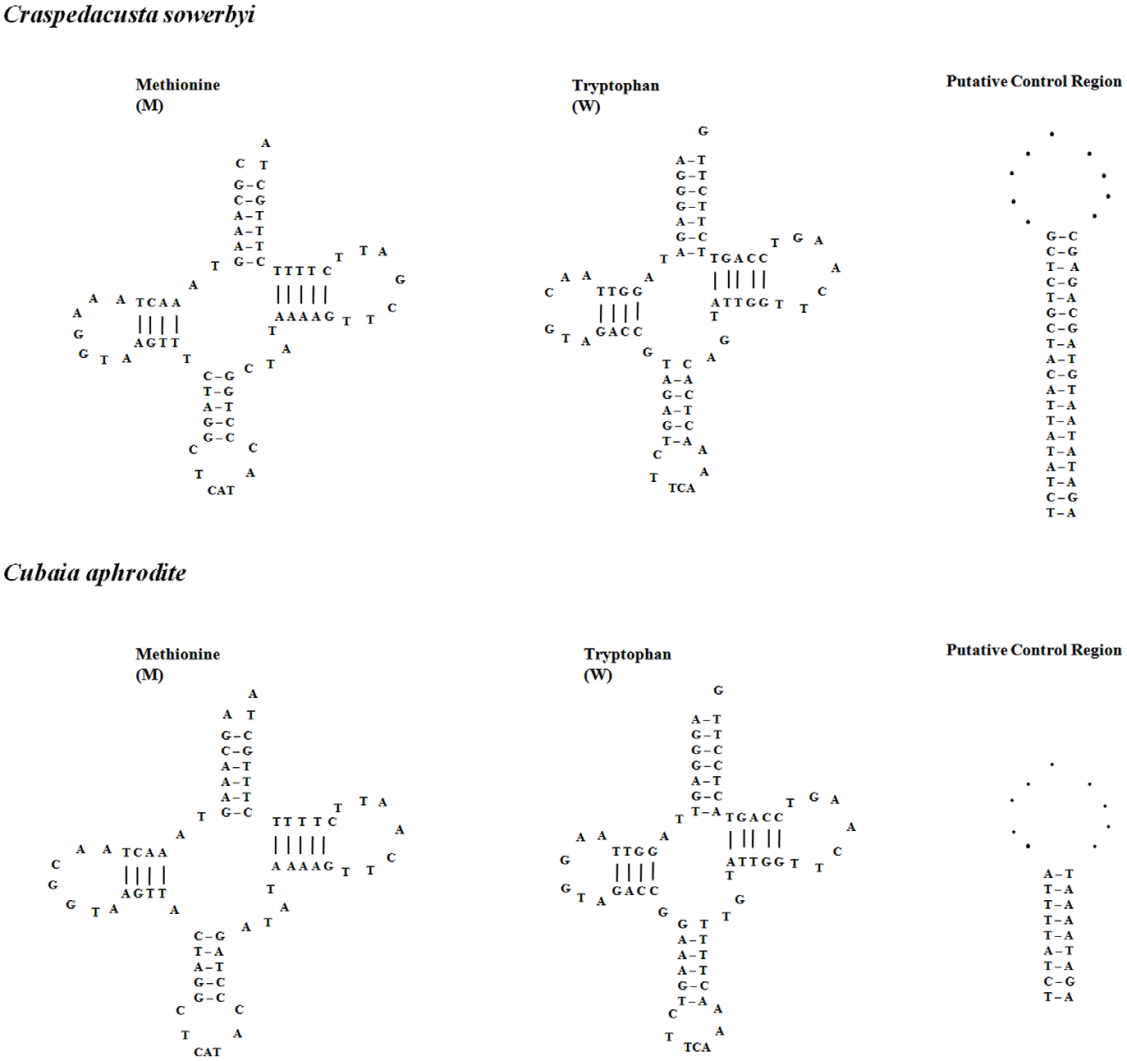
Secondary structure models predicted for tRNA and intergenic putative control region of *C. sowerbyi* and *C. aphrodite*.

Like many other cnidarians studied to date, *C. sowerbyi* mtDNA possessed only two tRNA genes for methionine (trnM; CAU) and tryptophan (trnW; UCA), and two rRNA genes coding for a large and a small subunit rRNA (*rnl* and *rns*). The nucleotide composition of RNA genes was slightly AT-richer than that of the protein coding regions. The sequences of *C. sowerbyi trnM* (*cau*) and *trnW* (*uca*) were both 71 bp in size as same as in *C. aphrodite*, which were well conserved with high similarity in cnidarians (Table 2) [7]. Inferred secondary structures of tRNAs in *C. sowerbyi* and *C. aphrodite* mtDNAs were shown in Fig. 2. The *C. sowerbyi* small and large subunit ribosomal RNA, *rns* and *rnl*, were 995 and 1,817 bp in size, respectively, similar to their counterparts in other cnidarians. These two genes were located more than 7 kilobases apart in *C. sowerbyi* mtDNA and had opposite transcriptional polarities.

### Intergenic Regions and Terminal Sequences

Intergenic regions constituted less than 1%, with 157 bp in total, in the *C. sowerbyi* mtDNA genome; the largest of these (60 bp) existed between *cox1* and *cox2*, which appeared coincidentally with the change in transcriptional polarity of *C. sowerby* mtDNA genes. As shown in Fig. 2, this AT-rich (68.3%) region can be folded into a conserved stem-loop motif with 19 bp in the stem; while in *C. aphrodite* mtDNA, a conserved stem-loop motif with only 9 bp in the stem was also discovered. Similar AT-rich regions were detected in other medusozoans [7]. This region was named as putative control region, and may be involved in the control of mtDNA transcription and/or replication [7].The second largest non-coding region was located upstream of *cox3*, with only 34 bp in size. The scarcity of non-coding nucleotides in the mitochondrial genome of *C. sowerbyi* was similar to the mtDNA of other reported medusozoans.

In addition to non-coding intergenic regions, non-coding flanking regions were present in the mitochondrial genome of *C. sowerbyi*. The identical inverted terminal repeats (ITRs), in which the sequence at one end of a determined segment is repeated in an inverted orientation at the other end, occurred on the *C. sowerbyi* linear mtDNA ends, as in other medusozoans [7], [8], [9], [10], [11]. The ITR region contained 435 bp, including a part of 3′ end of *cob* (59 bp). In the mtDNA of *A. aurita*, only 20 bp of the 3′ end of *cob* was included in the ITR region. The ITRs of *C. sowerbyi* lacked any obvious potential secondary structure and did not show any significant similarity to known sequences. In addition to *C. sowerbyi*, ITRs have determined for other four medusozoans: *H. oligactis*, *H. magnipapillata*, *A. aurita*, and *A. moseri*. They possessed feature of organelle telomeres and contained subtelomeric regions of fragmented and sometimes duplicate genes [10]. Though poorly understood, ITRs, together with the two ORFs of *polB* and *ORF314*, were suggested as remnants of a linear plasmid which invaded the mitochondrial genomes of the last common ancestor of Medusozoa and was involved in the mechanism of replication and maintenance for linear chromosomes similar to the type III linear mtDNA as found in yeasts, linear plasmids, and adenoviral DNA [7], [11].

### Phylogenetic Analysis

**Figure 3:**
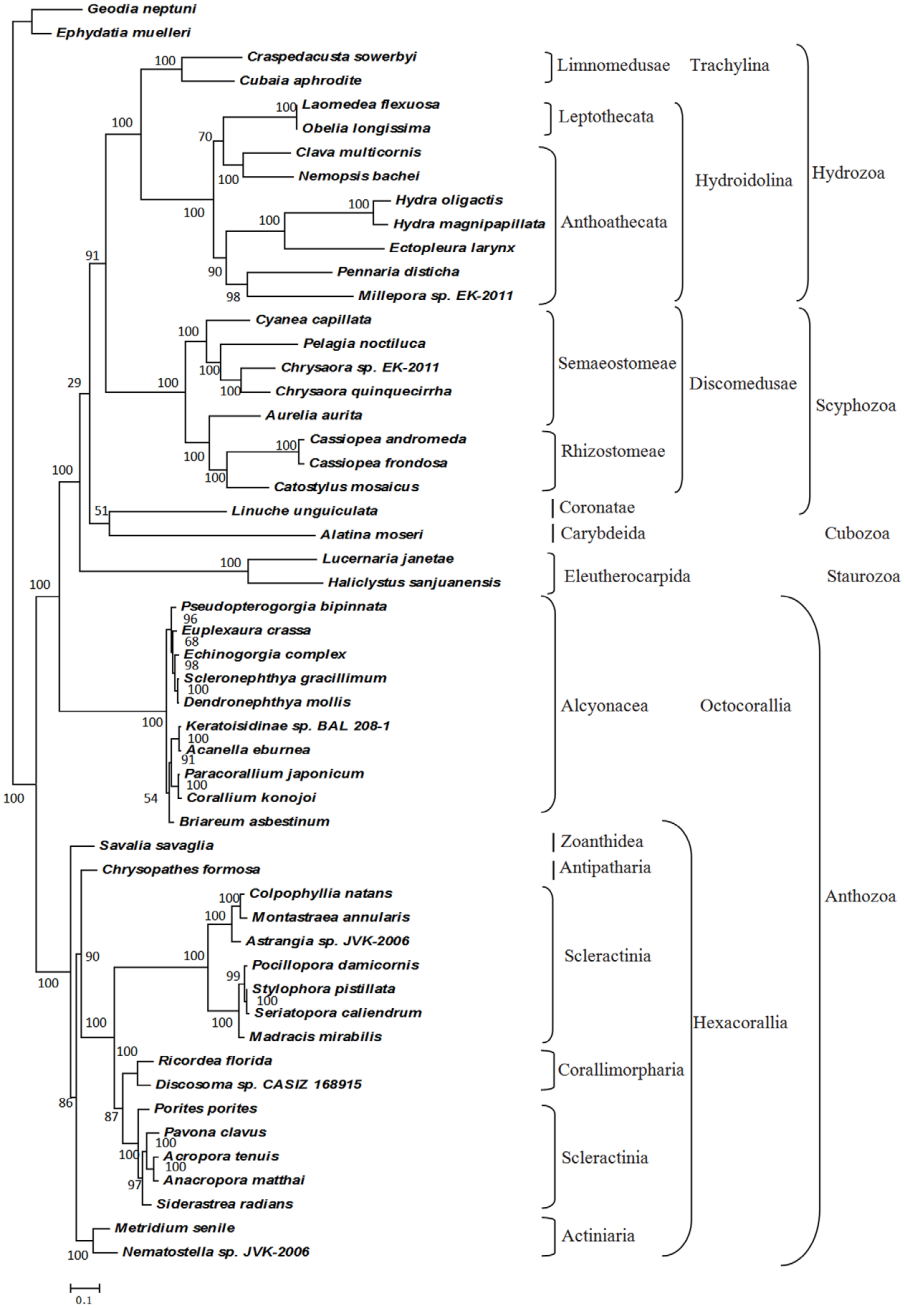
Phylogenetic analyses by ML methods based on mitochondrial inferred amino acid data. ML tree obtained from the analysis of inferred amino acids of 13 energy pathway protein genes under the MTMAM model. The branch support values for each node are shown as ML bootstrap percentage. Scale bars indicate number of changes per site.

The acquisition of a complete sequence of *C. sowerbyi* mtDNA in this study, as well as additional cnidarian mtDNA from GenBank, allowed us to investigate the phylogenetic relationship at the level of orders within the phylum Cnidaria. Maximum likelihood (ML) analyses of nucleotide and inferred amino acids data were conducted based on 13 energy pathway protein genes in 51 cnidarian species, with two sequences from species of the Demospongia as the outgroup. The tree topologies derived from phylogenetic trees based on nucleotide (Fig. S1) and inferred amino acids (Fig. 3) data were substantially congruent with just a few variations. As shown in Fig. 3, the Cnidaria appeared to be monophyletic, but was divided into two primary groups, with one group containing Medusozoa and octocorallian Anthozoa, and the other the hexacorallian Anthozoa.

In the subclass Hexacorallia, scleractinian corals were reconstructed as a paraphyletic group that included naked corals *Ricordia* and *Discosoma*. This result was consistent with the phylogenetic analysis (based on only mitochondrial amino acids data) by Medina et al., who deduced skeleton loss in naked corals [23]. The phylogenetic relationships found for octocorals in the present study are identical to those reported previously except that the scleraxonian *Briareum asbestinum* was placed at the base of Octocorallia in Park’s analyses [6]. Our analyses placed *B. asbestinum* as sister group of the other two scleraxonians plus two calcaxonians with weak support values, and the sampled octocorals formed two obvious clades.

All medusozoans here formed a clade containing species from all the four classes, Hydrozoa, Cubozoa, Scyphozoa, and Staurozoa. Staurozoa was the sister group of all other medusozoans (based on inferred amino acids data; Fig. 3). Interestingly, the only one cubozoan *Alatina moseri* and the only one coronate scyphozoan *Linuche unguiculata* fell into a clade, with weak support, which was then the sister group of other scyphozoans and hydrozoans, also with a low bootstrap support based on amino acids data (Fig. 3). Based on nucleotide data, cubozoan, staurozoans, and coronate scyphozoan formed a complex clade, and it was hard to determine the phylogenetic relationships exactly. Furthermore, discomedusan scyphozoans fell into monophyletic group, being a sister group with all hydrozoans. It appeared that Scyphozoa was paraphyletic. Semaeostomeae was reconstructed as paraphyletic, with the jellyfish *A. aurita* being more closely related to the Rhizostomeae than to the Semaeostomeae.

In the class Hydrozoa, the trachyline *C. sowerbyi* and *C. aphrodite* formed a clade, which was in turn a sister group to the monophylectic Hydroidolina (Fig. 3 and Fig. S1). Species in the Hydroidolina formed two clades, one clade containing species from Aplanulata and Capitata, while the other having species from Filifera III, Filifera IV and Leptothecata. In the phylogentic trees, Anthoathecata, consisting of Aplanulata, Capitata and Filifera III-IV, is paraphyletic with respect to leptothecates. Aplanulata containing *Hydra* and *Ectopleura larynx* was the sister group of non-Aplanulata Capitata.

## Discussion

In this study, we present the complete mtDNA sequence of the trachyline *C. sowerbyi*. Its mtDNA possessed the typical characteristics as reported for some other medusozoans: (a) a linear mtDNA molecule [3], [4]; (b) AMGO as identical to the gene order found in *A. aurita* [7], [11]; (c) a relatively compact genomic organization lacking introns [7]–[11]; (d) the presence of only two tRNA genes, *trnM* and *trnW* [24]; (e) flanking ITRs at the ends of the linear mtDNA [7], [8], [9], [10], [11]; (f) two extra ORFs of *polB* and *ORF314* at one end [7], [10], [11].

Thus far, only two mitochondrial genomes were determined in the order of Limnomedusae, with one from the marine species *C. aphrodite* and another from the freshwater jellyfish *C. sowerbyi*. The mtDNA sequences of these two species show high similarity with each other, especially in the presence of same gene order and the closest relationship in the phylogenetic tree. The AMGO and the two ORFs similar to *polB* and *ORF314* were found in mitogenomes of limnomedusans, as reported in staurozoans and discomedusan scyphozoans [7], [11]. However, the mitochondrial genomes of hydroidolinans have at least three gene orders similar to AMGO and lack the two ORFs [7], [8], [9]. Like other hydrozoans, Limnomedusae has a biphysic life history. It consists of a planula stage developing into a benthic polyp and a medusa stage developing via the entocodon. It appears to be easily characterized by two symplesiomorphies, i.e., ecto-endodermal statocysts and a life cycle that includes a polyp stage, shared with other members of Trachylina and Hydroidolina, respectively [2], [25]. Limnomedusae was once classified into a group named Hydroidomedusa (including Anthomedusae, Laingiomedusae, Leptomedusae, Limnomedusae, and Siphonophorae), which was characterized with a polyp stage budding medusae through a medusary nodule [26]. In the cladistic analysis based on 87 morphological and life history characters of medusozoans by Marques and Collins [1], Limnomedusae was identified as either the earliest diverging hydrozoan lineage or as the basal group of either Trachylina or Hydroidolina. However, it was eventually placed at the base of the subclass Trachylina rather than among the hydroidolinan groups according to the molecular data of mitochondrial 16 S and nuclear small and large subunit ribosomal genes [2], [5], [27], [28]. Our ML analyses based on the 13 energy pathway protein genes from mtDNA placed the limnomedusans at the base of Hydrozoa. Considering limnomedusans possess the gene order of AMGO and the shared ancestral characters of Trachylina and Hydroidolina, together with phylogenetic analyses in previous studies, we suggest that Limnomedusae be the earliest diverging lineage among Hydrozoa. However, this suggestion needs to be further examined by analyzing more mitogenomes from other hydrozoans, especially from other trachyline hydrozoans (Narcomedusae, Trachymedusae and Actinulida).

The complete mtDNA sequence of *C. sowerbyi* and other recently determined mtDNA of medusozoans allow us to analyze the phylogenetic relationship and mitochondrial evolution in Cnidaria, especially in Medusozoa. Generally, our findings in the ML trees were largely congruent with the previous reports based on mitochondrial and/or nuclear data [2], [5], [6], [7], [8], [29], [30]. As indicated in previous studies also employing mitogenomic data, the paraphyly of Anthozoa is further confirmed in the present analyses with octocorallians being closer to the medusozoans than to the hexacorallians [6], [8]. The evolutionary positions of the four medusozoan classes showing in our ML trees, were largely consistent with the speculation based on mitogenome organizations [7] and the phylogenetic analyses based on nuclear and mitochondrial ribosomal data [2], [5] in general. The monophyletic clade of hydrozoans was clustered as a sister group to the monophyletic discomedusan Scyphozoa. Hydrozoa appears to be composed of two well supported groups, Hydroidolina and Trachylina (Limnomedusae). In the group of Hydroidolina, our ML analyses provided strong support for a clade of capitates to the exclusion of aplanulate taxa, which supported the previous studies that divided the Capitata into clades of Aplanulata and non-Aplanulata Capitata [30]. As the newly recognized class in Medusozoa, Staurozoa was removed from Scyphozoa by Marques and Collins [1] for its discrete history and features correspondingly distinct from other cnidarian classes. In the present study we recognize that Staurozoa should be the sister group of all other medusozoans as shown in the ML tree based on amino acids with 100 bootstrap percentage. Previous studies that employed other molecular data [2], [5], [7] and morphological evidence [31] also generated similar hypothesis. Cubozoans were removed from Scyphozoa and given ‘class’ status by Werner [32]. However, the position of Cubozoa within Medusozoa has been rather contentious, with some favoring a hydrozoan affinity [33], [34] and others a closer relationship to scyphozoans [1], [35], [36], [37]. Molecular analyses based on large and small ribosomal genes placed Cubozoa and Scyphozoa at a clade that was the sister group of Hydrozoa [2]. When referred to the mitogenome, the only cubozoan representative with eight linear mitochondrial chromosomes fell into a clade with the only representative of coronate scyphozoans in the phylogenetic analysis based on amino acids. Our analyses had Cubozoa and coronate Scyphozoa forming a clade that was the sister group of the rest scyphozoans and hydrozoans, and it seems that Coronatae is not as close to the rest scyphozoans as we supposed. However, the very limited species from these interesting groups make the hypothesis not compelling enough and further studies including more species need to be carried out.

## Materials and Methods

### Ethics Statement

No specific permits were required for collection the samples of medusae of Craspedacusta for scientific intention in China. The sampling location was not privately-owned or protected in any way, and no specific permissions were required for the location. All the field studies did not involve endangered or protected species.

### Collection of Medusae and Isolation of DNA

Medusae of *Craspedacusta* were collected from a lake (30° N 29′ 37.21′′, 114°E 43′ 40′′) in Wuhan, Hubei province, China. Specimens were identified as *C. sowerbyi* according to the description by Jankowski [12] and the sequences alignments of internal transcribed spacers and cytochrome oxidase subunit I (COI). Individual medusae were washed in double-distilled water before being stored in 90% ethanol. Specimens were then soaked for 1 day in TE buffer (pH 8.0) to remove ethanol before the isolation of DNA. Genomic DNA was extracted using a standard sodium dodecylsulfate-proteinase K procedure described by Sambrook et al. [38].

### Polymerase Chain Reaction (PCR) and Genome Sequencing

Primers were designed to match highly conserved regions of animal mtDNA genes and were used to amplify short fragments from the conserved genes of *cox1*, *rnl*, *nad4* [11], *cox3* and *cob* [39]. Then, specific primers (Table S1) were designed and used with Takara® LA PCR kits to amplify mtDNA between *rnl* and *cob* in several PCR reactions. The peripheral regions of the molecule were amplified using the hiTAIL-PCR method [40] with specific primers and anchor primers (Table S1). PCR products were cloned into pGEM-T vector (Promega, USA) and sequenced. *C. sowerbyi* mtDNA sequences are available via Genbank accession number JN593332.

### Gene Annotation and Analysis

Transfer RNA (tRNA) genes in the mtDNA were identified by the tRNAscan-SE program [41]; other genes were identified by similarity searches on the NCBI web site using the BLAST program. The secondary structures of tRNA-Met and tRNA-Trp were deduced by comparison with their homologues in *A. aurita* and *H. oligactis*.

For analysis of sequence similarities, individual genes were aligned with ClustalW 1.82 [42] using default parameters; alignments were manually adjusted based on either amino acid alignments (for protein coding genes) or inferred rRNA or tRNA secondary structures (for RNA-coding genes). Sequence identities were calculated using local scripts based on BioPerl modules [43]. Codon usage was calculated with the CUSP program in the EMBOSS package [44].

### Phylogenetic Analyses

Phylogenetic analyses were conducted on both amino acid and nucleotide sequences of the coding regions of 13 genes related to respiration and oxidative phosphorylation. The inferred sequences were derived from the GenBank files listed in Table S2.

Fifty-one mtDNA coding sequences of the species in Cnidaria and two sequences of the species in Demospongia were used for phylogenetic analysis based on nucleotides and inferred amino acids data, respectively. Mitochondrial coding sequences were aligned based on amino acid alignments using CodonAlign [45] and recoded using the “neutral transition model” coding strategy [46]. Amino-acid sequences of individual proteins were aligned three times with ClustalW 1.82 [41] using different combinations of opening/extension gap penalties: 10/0.2 (default), 12/4 and 5/1. The three alignments were compared using SOAP [47], and only positions that were identical among them were included in phylogenetic analyses. A maximum likelihood (ML) search was performed for the best tree using RAxML 2.2.3 [48]. The program ProtTest version 1.3 suggested mtMAM model of sequence evolution [49], [50] for the ML analyses of the amino acid data, while the program of ModelTest suggested that a GTR model with gamma [51] was the best for ML analyses of the nucleotide data. One hundred replicates for rapid bootstrap analyses [52] were also performed using RAxML, and a 50% majority rule consensus was calculated to determine the support values for each node.

## Supporting Information

Figure S1 **Phylogenetic analyses by ML methods based on mitochondrial nucleotide data.** ML tree obtained from the analysis of aligned and recoded nucleotides of 13 energy pathway protein genes under the GTR+GAMMA model. The branch support values for each node are shown as ML bootstrap percentage. Scale bars indicate number of changes per site.
(TIF)

Table S1 **Primers for amplification of** ***C. sowerbyi*** **.**
(DOC)

Table S2 **List of GenBank accession numbers for mitochondrial genomes.**
(DOC)
