# Supplementary figures and images for: Mitochondrial Genome of the Freshwater Jellyfish Craspedacusta sowerbyi and Phylogenetics of Medusozoa
Source: PLoS One. 2012 Dec 11;7(12):e51465. doi: 10.1371/journal.pone.0051465 (PMC3519871; doi:10.1371/journal.pone.0051465)

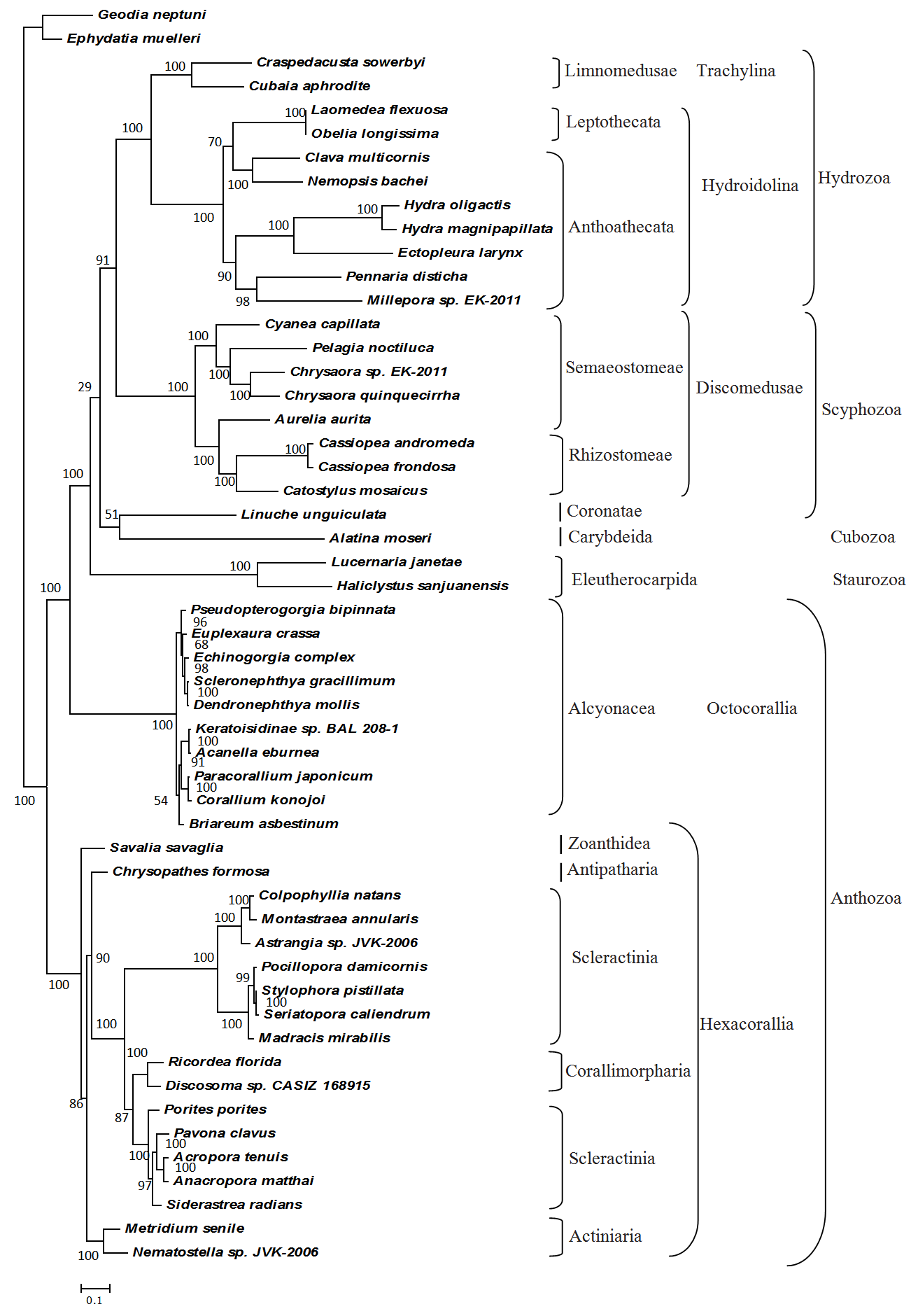

Supplement: Figure S1 — Phylogenetic analyses by ML methods based on mitochondrial nucleotide data. ML tree obtained from the analysis of aligned and recoded nucleotides of 13 energy pathway protein genes under the GTR+GAMMA model. The branch support values for each node are shown as ML bootstrap percentage. Scale bars indicate number of changes per site. (TIF) [file pone.0051465.s001.tif]
