# Supplementary material for: Mitochondrial Genome of the Freshwater Jellyfish Craspedacusta sowerbyi and Phylogenetics of Medusozoa
Source: PLoS One. 2012 Dec 11;7(12):e51465. doi: 10.1371/journal.pone.0051465 (PMC3519871; doi:10.1371/journal.pone.0051465)
Supplement: Table S1 — Primers for amplification of C. sowerbyi . (DOC) [file pone.0051465.s002.doc]

**Table S1** Primers for amplification of *C. sowerbyi*.

| Primers | Sequences（5’-3’） | References |
| --- | --- | --- |
| Cs16sF | GATGTCGACCTCTGACCGTGATAATGT | Present study |
| Cs16SR | GATGCGGCCGCGATTACGCTGTTATCCCTA | Present study |
| CsCOIF | ATGGCGGCCGCATAATGTCATCGTCACCG | Present study |
| CsCOLR | GATGTCGACGGGGTCAAAGAAAGTGGT | Present study |
| COIIIF | TGGTGGCGAGATGTKKTNCGNGA | 38 |
| COIIIR | ACWACGTCKACGAAGTGTCARTATCA | 38 |
| nad4F | CCKAARGCYCAYGTKGARGCYCC | 10 |
| nad4R | GARGAWCAKAWWCCRTGAGCAATYAT | 10 |
| CytbF | GGWTAYGTWYTWCCWTGRGGWCARAT | 38 |
| CytbR | GCRTAWGCRAAWARRAARTAYCAYTCWGG | 38 |
| W07-1 | AGGTCGCCCCAACCAAACTACCACTCT | Present study |
| W29-1 | AGTTTCTGACTCCTCCCTCCTGCCCT | Present study |
| W30-3 | GCAAGAGGGGGATAGACCGTCCAACC | Present study |
| W37-3 | GAAGAACTGCCGTGTTTAAGAGGGGT | Present study |
| W36-3 | GGGTCGGTGTTTTTTGTCGCTACTGG | Present study |
| W40-1 | CTGAAGCACAAGGAAAGAGGGGGAAC | Present study |
| W38-3 | CAAAGTTGGTTATGACCGTGGCTCCC | Present study |
| W41-3 | GAAGGCTTAGTCGCTTCGCTCTTTCT | Present study |
| W39-1 | TATGAGGAGGATTCAGCGTTAGCGGA | Present study |
| W39-2 | ACGATGGACTCCAGTCCGGCCTGAGTCTATTGGTATTCTACGCCCCG | Present study |
| W39-3 | ATTCCAGCCAACCCCTTAGTGACCCC | Present study |
| W32-1 | GGTAACTCTGACCGTGATAATGTAGCGA | Present study |
| W32-2 | ACGATGGACTCCAGTCCGGCCGGAGTGCATCGAGAGTGGTAGTTTGG | Present study |
| W32-3 | GTAGTTTGGTTGGGGCGACCTTCTTCT | Present study |
| W33-1 | TATACATTCAAACCCCCACCCACGCA | Present study |
| W33-2 | ACGATGGACTCCAGTCCGGCCCAAATCCAACTTCCCATACCGAGCCT | Present study |
| W33-3 | TGTCACAAATCGCCCCATCTCCTTCT | Present study |
