# Supplementary material for: Mitochondrial Genome of the Freshwater Jellyfish Craspedacusta sowerbyi and Phylogenetics of Medusozoa
Source: PLoS One. 2012 Dec 11;7(12):e51465. doi: 10.1371/journal.pone.0051465 (PMC3519871; doi:10.1371/journal.pone.0051465)
Supplement: Table S2 — List of GenBank accession numbers for mitochondrial genomes. (DOC) [file pone.0051465.s003.doc]

**Table S2.** List of GenBank accession numbers for mitochondrial genomes.

| Species | Accession No. |
| --- | --- |
| [**Porifera**](http://www.ncbi.nlm.nih.gov/Taxonomy/Browser/wwwtax.cgi?mode=Info&id=6040&lvl=3&lin=f&keep=1&srchmode=1&unlock); [**Demospongiae**](http://www.ncbi.nlm.nih.gov/Taxonomy/Browser/wwwtax.cgi?mode=Tree&id=6042&lvl=3&lin=f&keep=1&srchmode=1&unlock) | |
| *Geodia neptuni* | NC_006990 |
| *Ephydatia muelleri* | EU237481 |
| **Hydrozoa, Trachylina, Limnomedusae** | |
| *Craspedacusta sowerbyi* | JN593332 |
| *Cubaia aphrodite* | JN700942 |
| **Hydrozoa, Hydroidolina, Leptothecata** | |
| *Laomedea flexuosa* | JN700945 |
| *Obelia longissima* | JN700948 |
| **Hydrozoa, Hydroidolina, Anthoathecata** | |
| *Clava multicornis* | JN700935 |
| *Nemopsis bachei* | JN700947 |
| *Hydra oligactis* | NC_010214 |
| *Hydra magnipapillata* | NC_011220, NC_011221 |
| *Ectopleura larynx* | JN700938 |
| *Pennaria disticha* | JN700950 |
| *Millepora sp.* EK-2011 | JN700943 |
| **Scyphozoa, Discomedusae, Semaeostomeae** | |
| *Cyanea capillata* | JN700937 |
| *Pelagia noctiluca* | JN700949 |
| *Chrysaora sp.* EK-2011 | JN700941 |
| *Chrysaora quinquecirrha* | HQ694730 |
| *Aurelia aurita* | NC_008446 |
| **Scyphozoa, Discomedusae, Rhizostomeae** | |
| *Cassiopea andromeda* | JN700934 |
| *Cassiopea frondosa* | JN700936 |
| *Catostylus mosaicus* | JN700940 |
| **Scyphozoa, Coronatae** | |
| *Linuche unguiculata* | JN700939 |
| **Cubozoa, Carybdeida** | |
| *Alatina moseri* | JN642330, JN642332, JN642334, JN642336, JN642337, JN642340, JN642342, JN642344 |
|  |  |
| **Staurozoa, Eleutherocarpida** | |
| *Lucernaria janetae* | JN700946 |
| *Haliclystus sanjuanensis* | JN700944 |
| **Anthozoa, Octocorallia, Alcyonacea** | |
| *Pseudopterogorgia bipinnata* | NC_008157 |
| *Euplexaura crassa* | HQ694728 |
| *Echinogorgia complexa* | HQ694727 |
| *Scleronephthya gracillimum* | GU047879 |
| *Dendronephthya mollis* | HQ694725 |
| *Keratoisidinae sp*. BAL 208-1 | NC_010764 |
| *Acanella eburnea* | NC_011016 |
| *Paracorallium japonicum* | NC_015405 |
| *Corallium konojoi* | NC_015406 |
| *Briareum asbestinum* | NC_008073 |
| **Anthozoa, Hexacorallia, Zoanthidea** | |
| *Savalia savaglia* | NC_008827 |
| **Anthozoa, Hexacorallia, Antipatharia** | |
| *Chrysopathes formosa* | NC_008411 |
| **Anthozoa, Hexacorallia, Scleractinia** | |
| *Colpophyllia natans* | NC_008162 |
| *Montastraea annularis* | NC_007224 |
| *Astrangia sp*. JVK-2006 | NC_008161 |
| *Pocillopora damicornis* | NC_009797 |
| *Stylophora pistillata* | NC_011162 |
| *Seriatopora caliendrum* | NC_010245 |
| *Madracis mirabilis* | NC_011160 |
| **Anthozoa, Hexacorallia, Corallimorpharia** | |
| *Ricordea florida* | NC_008159 |
| *Discosoma sp*. CASIZ 168915 | NC_008071 |
| **Anthozoa, Hexacorallia, Scleractinia** | |
| *Porites porites* | NC_008166 |
| *Pavona clavus* | NC_008165 |
| *Acropora tenuis* | NC_003522 |
| *Anacropora matthai* | NC_006898 |
| *Siderastrea radians* | NC_008167 |
| **Anthozoa, Hexacorallia, Actiniaria** | |
| *Metridium senile* | NC_000933 |
| *Nematostella sp*. JVK-2006 | NC_008164 |
